# Distribution and Risk Factors for *Plasmodium* and Helminth Co-infections: A Cross-Sectional Survey among Children in Bagamoyo District, Coastal Region of Tanzania

**DOI:** 10.1371/journal.pntd.0003660

**Published:** 2015-04-02

**Authors:** Nahya Salim, Stefanie Knopp, Omar Lweno, Ummi Abdul, Ali Mohamed, Tobias Schindler, Julian Rothen, John Masimba, Denis Kwaba, Alisa S. Mohammed, Fabrice Althaus, Salim Abdulla, Marcel Tanner, Claudia Daubenberger, Blaise Genton

**Affiliations:** 1 Bagamoyo Research and Training Centre, Ifakara Health Institute, Bagamoyo, United Republic of Tanzania; 2 Department of Pediatrics and Child Health, Muhimbili University Health and Allied Sciences (MUHAS), Dar es Salaam, United Republic of Tanzania; 3 Department of Epidemiology and Public Health, Swiss Tropical and Public Health Institute, Basel, Switzerland; 4 University of Basel, Basel, Switzerland; 5 Department of Life Sciences, Wolfson Wellcome Biomedical Laboratories, Natural History Museum, London, United Kingdom; 6 Department of Medical Parasitology and Infection Biology, Swiss Tropical and Public Health Institute, Basel, Switzerland; 7 Department of Ambulatory Care and Community Medicine, Infectious Disease Service, Lausanne University Hospital, Lausanne, Switzerland; Mahidol University, THAILAND

## Abstract

**Background:**

*Plasmodium* and soil transmitted helminth infections (STH) are a major public health problem, particularly among children. There are conflicting findings on potential association between these two parasites. This study investigated the *Plasmodium* and helminth co-infections among children aged 2 months to 9 years living in Bagamoyo district, coastal region of Tanzania.

**Methods:**

A community-based cross-sectional survey was conducted among 1033 children. Stool, urine and blood samples were examined using a broad set of quality controlled diagnostic methods for common STH (*Ascaris lumbricoides*, hookworm, *Strongyloides stercoralis*, *Enterobius vermicularis*, *Trichuris trichura*), schistosoma species and *Wuchereria bancrofti*. Blood slides and malaria rapid diagnostic tests (mRDTs) were utilized for *Plasmodium* diagnosis.

**Results:**

Out of 992 children analyzed, the prevalence of *Plasmodium* infection was 13% (130/992), helminth 28.5% (283/992); 5% (50/992) had co-infection with *Plasmodium* and helminth. The prevalence rate of *Plasmodium*, specific STH and co-infections increased significantly with age (p < 0.001), with older children mostly affected except for *S*. *stercoralis* monoinfection and co-infections. Spatial variations of co-infection prevalence were observed between and within villages. There was a trend for STH infections to be associated with *Plasmodium* infection [OR adjusted for age group 1.4, 95% CI (1.0–2.1)], which was more marked for *S*. *stercoralis* (OR = 2.2, 95% CI (1.1–4.3). Age and not schooling were risk factors for *Plasmodium* and STH co-infection.

**Conclusion:**

The findings suggest that STH and *Plasmodium* infections tend to occur in the same children, with increasing prevalence of co-infection with age. This calls for an integrated approach such as using mass chemotherapy with dual effect (e.g., ivermectin) coupled with improved housing, sanitation and hygiene for the control of both parasitic infections.

## Author Summary

Parasitic infectious agents rarely occur in isolation and multiparasitism is a norm specifically in children living in endemic areas of Tanzania. We studied the pattern and predictors of *Plasmodium* and STH co-infections in rural Bagamoyo district, coastal region of Tanzania. Parents/guardians of healthy children aged 2 months to 9 years who were willing to participate in the study were invited from the community. Stool, urine and blood were examined for helminth and *Plasmodium* parasites. We found that children aged above five years and those who are not schooling had the greatest burden of co-infection with *Plasmodium* and helminth parasites. The risk of being co-infected with *Plasmodium* increased with age with all the common types of STH isolated (*E*. *vermicularis*, hookworm and *S*. *stercoralis*). Younger children had a significantly higher risk of having *Plasmodium* when co-infected with *S*. *stercoralis*. Integrated control approaches including health education, environmental sanitation and hygiene, novel chemoprophylaxis as well as long lasting Impregnated Nets (LLINs) distributions should be implemented considering the pattern and types of infections within the area in order to interrupt transmission of both parasites among young and school-aged children.

## Introduction

Parasitic infections such as *Plasmodium* present a major public health problem among children in Africa [1,2] and its coexistence with Soil Transmitted Helminth (STH) infections is common [3,4]. Multipasitism is a norm among children in developing countries including United Republic of Tanzania [5,6]. It is defined as a concurrent infection in a single host with two or more species whereas monoinfection consists of only one infection from a single species [7]. Variety of environmental and host related factors can influence the structure and dynamics of the parasite communities which make up these multiple infections [8–10]. These conditions include poverty, environmental contamination with infected faeces containing helminth eggs, water bodies, lack of effective preventive measures [11] and immunity of the host. In addition, overlap of *Plasmodium* infection and other pathogens depends on the conditions that favour multiple parasitic species survival and transmission such as exposure related risk and within host interactions between co-infecting species [3,11,12]. There is mounting evidence indicating that helminth infections increase susceptibility to *Plasmodium* infection [13–15]. On the other hand, some studies showed that specific STH like *Ascaris lumbricoides* are protective against *Plasmodium* disease and its severe manifestations [16]. Previous epidemiological studies have shown coexistence of *Plasmodium* and helminth infections with spatial heterogeneity in their distribution [3,4,6,17]. In Tanzania, cross-sectional surveys conducted among school and preschool children showed that multiple parasitic infections are common [5,6,17]. Study by Kinung’hi et al showed that the prevalence of malaria parasites tended to increase with increasing number of co-infecting helminth species as compared to helminth free children although the difference was not statistically significant [6].

In Tanzania, global strategies to control malaria are being conducted by the National malaria control program (NMCP) via Long Lasting Insecticide Impregnated Nets (LLINs), Intermittent Preventive Treatment in Pregnancy (IPTp) and prompt treatment with artemether/lumefantrine. The helminth control is done via mass drug administration (MDA), chemotherapy based morbidity control campaigns. The planning for prevention and control program are designed to focus on a single infection approach despite occurrence of co-infections [18]. There is an underestimation of the burden of infection and lack of understanding how these parasitic infections interact [18]. This underlines the importance of investigating the epidemiology of co-infections in different geographical locations where different pattern of infections are expected. In the present study we aimed to determine the relation between *Plasmodium* and STH co-infections among children aged 2 months to 9 years living in Bagamoyo district, coastal region of Tanzania. Knowledge of the magnitude and on the common risk factors for co-infections should guide the development of focused integrated control programs targeting multiple infections endemic in each country.

## Materials and Methods

Reporting of the study follows STROBE checklist (Strengthening the Reporting of Observational studies in Epidemiology) [19].

### Ethics statement

The study was conducted under the IDEA study protocol which was approved by the institutional review boards of the Swiss Tropical and Public Health Institute (Swiss TPH; Basel, Switzerland) and the Ifakara Health Institute (IHI; Dar es Salaam, United Republic of Tanzania). The ethical approval for the conduct of the study was granted by the Ethikkomission beider Basel (EKBB; Basel, Switzerland; reference number: 257/08) and the National Institute for Medical Research of Tanzania (NIMR; Dar es Salaam, United Republic of Tanzania; reference number: NIMR/HQ/R.8a/Vol. IX/1098).

The local district, community, school teachers and health authorities were informed during sensitization meetings about the purpose, procedures, risk and benefits of the study prior to the start. Written informed consent was obtained from the parents/guardians of children prior to study procedures after explaining them to the group. Illiterate parents/ guardians were asked to bring witness who participated within the discussion prior to obtaining their thumbprints and witness signature. Participants infected with helminth and/or malaria or other medical conditions received appropriate treatment/referral according to the national treatment guidelines of Tanzania.

### Study area

Bagamoyo is a district in the coastal region of Tanzania where Ifakara Health Institute (IHI) through its branch, Bagamoyo Research and Training Centre (BRTC) works in close collaboration with the Bagamoyo District hospital (BDH) officials to ensure quality health care delivery using its research platforms. The BRTC study area covers about 1160 square kilometres. The eastern border of the study area is formed by the Indian Ocean, with the Ruvu river forming part of the western and northern borders. The area extends for approximately 7 km on either side of a road running westwards for 62 kilometres. To the south is an uninhabited forest reserve. According to the 2012 Tanzania National Census, the population of the Bagamoyo District was 311,740 which can be reached by dirt road throughout the year; all are within an hour drive [20].

The main rainy season is from March to May, with a second period from November to December, although occasional rain occurs at all times of the year. Average rainfall is 1200 to 2100 mm per year. There is year round grassland vegetation or subsistence agriculture throughout the study area. According to meteorological statistics the average temperature for the region is about 28°C. Majority of the people are either subsistence farmers who cultivate rice, maize and cassava, or fish from the sea or the Ruvu River and its tributaries. Agriculture employs 76% of the population [20].

The survey was conducted in the western rural area including hamlets within the villages of Kiwangwa, Msata, Mkange and Magomeni. The settlements are located about 20 to 60 km from Bagamoyo town. The inhabitants of the villages are mainly smallholder farmers engaged in food crop production such as pineapples, cassava, maize, vegetables and in fishing and salt mining. The prevalence of malaria within the western study area is still high compared to Bagamoyo town with seasonal variations secondary to malaria interventions through research and Tanzania National Malaria Control program (NMCP) [21]. Research on malaria drugs and vaccine have been conducted in Bagamoyo town since 2005 through IHI and its collaborative partners. The rural water supply is mostly from dams and ponds [22] highly contaminated with fecal coliform bacteria [23]. Eighty four percent of the communities have soil based latrines [22]. The latter resemble to simple pit latrines but without floor nor hygiene cover slab, nor lid covering the hole.

### Study design

The study is part of the IDEA project, an African-European Research initiative, funded by European community, with the aim of dissecting the immunological interplay between poverty related diseases (malaria, TB and HIV) and helminth infections[24]. The present community cross-sectional survey was conducted at the start of the IDEA malaria project to provide baseline data to inform further prospective immune-epidemiological studies of malaria infected individuals.

### Participant recruitment and sample collection

Study population included a random sample of healthy children as regarded by their parents/guardians, aged 2 months to 9 years inclusively, whose parents/guardians where informed about the study through Village Health Care Workers (VHCW) and agreed to come for screening at the meeting points. The villages were purposely selected based on the environmental conditions favouring both malaria and helminth survival and transmission. In the malaria arm, a sample size of 100 children with asymptomatic *Plasmodium* parasitemia was required. The malaria prevalence being 10% within the study area [25], we enrolled about ~1000 children from the community survey [26]. Standardized questionnaires were used to collect information on demographics, vital and clinical signs and symptoms to ensure that they were free of common diseases at that point in time. Parents or guardians where asked about interventions implemented within the Tanzanian National program, namely the use of long-lasting impregnated bednets (LLINs) and prior anti-helminth treatment. Participant recruitment and data collection was done between July 2011 and November 2012 covering an entire year and thus including seasonal variation [26].

All children had a finger prick to obtain about 1ml of blood which was collected in an Ethyl Diamine Tetra acetic Acid (EDTA) tube for malaria slide and full blood count which were performed at the main BRTC laboratory. Malaria rapid diagnostic test (SD BIOLINE, SD standard diagnostics, inc.Korea) and hemoglobin level using HemoCue hemoglobinometer (EKF diagnostic GmbH, Germany) were done in the field for inclusion/exclusion criteria and immediate management of the children with malaria and severe anemia.

Additionally, each participant was provided with i) two clean containers (100mls) for stool and urine samples ii) a plastic pocket with an adhesive tape (50 x 20mm) and a glass slide. All labelled with participant identification number. Parents/guardians were instructed on how to apply the adhesive tape and advised to collect sufficient amount of fresh stool and urine. The filled containers and adhesive tape slide were collected by the VHCW at a predefined meeting point in the village centre, the next day before noon and submitted to the Helminth Unit (HU) of the BRTC where all stool and urine samples were examined by experienced technicians.

### Diagnosis of *Plasmodium* infection

Thick and thin blood films were prepared, air dried and Giemsa stained for detection and quantification of malaria parasites according to the IHI laboratory Standard Operating Procedures (SOP). To detect malaria parasites, 200 fields were examined. Parasite density expressed per μl of blood was calculated by multiplying a factor of 40 to the number of parasites counted, assuming 8,000 leucocytes per μl of blood [27]. All slides were read by two independent qualified technicians. In case of discrepancy between two readers, a third reader was requested. The final result was the geometric mean of the two geometrically closest readings out of the three. For cases of positive/negative discrepancy the majority decision was adopted. If the test results were positive, the final one was then taken as the geometrical mean of the two positive results.

### Diagnosis of helminth infection

Duplicate Kato-Katz thick smear slides using a 41.7 mg template, adhesive tape slides, Baermann and FLOTAC methods were used to diagnose intestinal helminth [28]. Microhaematuria was examined using a dipstick (Hemastix; Siemens Healthcare Diagnostics, Eschborn, Germany) and for *S*. *haematobium* eggs by urine filtration (hydrophilic polycarbonate membrane filter; pore size 20 micron, diameter 13mm; Sterlitech, Kent, WA, United States of America). Binax NOW Filariasis rapid immunochromatic test (ICT) card (inverness medical professional diagnostics; ME; United States of America) was performed at the HU to detect *W*. *bancrofti* antigen using whole blood All Kato-Katz thick smear, adhesive tape and urine filtration slides were stored in boxes and 10% of slides re-examined for quality control by the senior experienced personnel after 3–6 months [28].

### Data management and statistical analysis

The helminth species specific results derived by each method were entered into an electronic data base using Microsoft ACCESS 2010. Double entry of the clinical and laboratory data was done using the DMSys software (FDA approved for ICH/GCP clinical trials). The two datasets were transferred into STATA format merged and cleaned. Data analysis was performed using STATA version 11.0 software (Stata Corp LP; College Station, Texas, USA). For duplicate Kato-Katz methods, the average of the two slides multiplied by a factor 24 was done to obtain egg per gram of stool (EPG). *S*. *stercoralis* and *E*. *vermicularis* intensity expressed as larvae counts and number of eggs counted respectively. Helminth infection intensity was categorized according to WHO criteria [29].

To investigate the relationship between *Plasmodium* and helminth, only the children who had both *Plasmodium* and helminth results were included. Table 1 defines the terms used in the analysis for easiness of interpretation. Geographical information system (arcgis 10) was used to map the distribution of *Plasmodium* and helminth monoinfections/co-infections among children in the coastal region of Bagamoyo. Harmonization of the identified hamlets within the studied villages was achieved using the known registered villages and hamlets/streets names from the Tanzania shape file (http://openmicrodata.wordpress.com/2010/12/16/tanzania-shapefiles-for-eas-villages-districts-and-regions/). Hamlets with low numbers (less than 10) were systematically merged to the nearest hamlet within the same village. Baseline characteristics were presented within age groups (children less than 3 years, preschool children aged 3–5 years and school-aged children from 6–9 years). The categorization was chosen to explore the age dependency variability considering the ongoing malaria and mass drug administration helminth programs with different approaches based on age, mainly focusing on under five and above five years of age. Crude odds ratios (ORs) including 95% confidence interval and p-values were calculated for variables potentially associated with infections/co-infections. To investigate risk factors, different models were explored with all *Plasmodium*, *Plasmodium* monoinfection, helminth mono- and mixed infections, and *Plasmodium* + helminth co-infections. The association between helminth species and *Plasmodium* infection was explored to further study co-infection patterns. Variables that were associated in bivariate analysis with a p-value level of < 0.05 were considered in multiple logistic regression models. The association between *Plasmodium* and helminth co-infections was subsequently investigated using negative binomial regression estimation after comparing the conditional means and variances of variables, all variances greater than means signifying over dispersion of data. To further investigate the age dependency relationship, Mantel—Haenszel stratified odds ratios (ORs) were conducted.

**Table 1:** Definitions of the terms.

Single helminth infection	Helminth monoinfection, one species only
Mixed helminth infection	More than one species of helminth
All helminth infections	Single, mixed helminth infection and co-infection
*Plasmodium* monoinfection*	*Plasmodium* species infection only
All *Plasmodium* infections	*Plasmodium* monoinfection and co-infection
Co-infection	*Plasmodium* and helminth co-infection
	*Plasmodium* and single helminth
	*Plasmodium* and Mixed helminth

*The term single *Plasmodium* was not used because only *P*. *falciparum* species is considered as the main cause of infection during this analysis [2].

A more detailed description and analysis of the helminth distribution is provided in the published paper [26].The present one focuses on the relationship of soil transmitted helminth (STH) and *Plasmodium* as prevalence of other helminth like *W*. *bancrofti* and *S*. *hematobium* were low among the studied children.

## Results

### Baseline characteristics of the study participants

**Fig 1:**
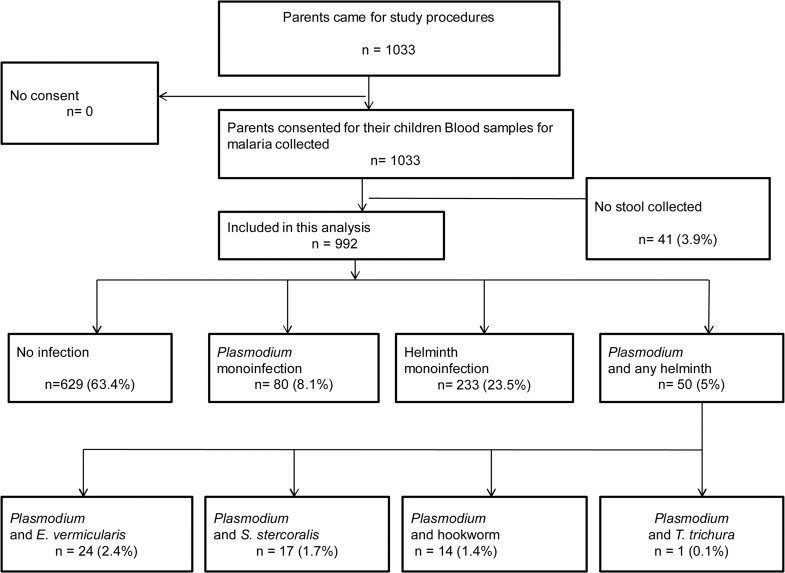
Flow of study participants and prevalence of *Plasmodium* and helminth infections.

A total of 1033 children were recruited. Of these, 41 (3.9%) did not submit stool samples which left 992 children as study analysis population (Fig 1). Demographic characteristics and intervention coverage are described in Table 2. Overall median age was 4.7 years with 25^th^ of 2.3 and 75^th^ quartiles of 6.5; 459 (46%) were represented by children above five years of age. Among children aged five years and above 185 (40.3%) were not schooling at the time of the survey. 494 (49.8%) were males. Eight hundred and twenty (82.7%) of the study population slept under a long lasting insecticide impregnated net (LLIN) the night before the survey. The parents/guardians reported use of albendazole and mebendazole past six months in 411 (41.4%) and 188 (18.9%) of their children respectively.

**Table 2:** Demographic characteristics and intervention coverage of study participants by age group.

Characteristics	< 3 years (n = 297)	3–5 years (n = 236)	> 5 years (n = 459)	Total (%) N = 992
Median age (25^th^–75^th^ Quartile)	1.5 (0.8–2.2)	4.1 (3.7–4.5)	6.7 (5.8–7.7)	4.7 (2.3–6.5)
**Gender**				
Male	162 (54.5)	118 (50.0)	214 (46.6)	494 (49.8)
Female	135 (45.5)	118 (50.0)	245 (53.4)	498 (50.2)
**Education level**				
Too young	290 (97.7)	181 (76.7)	61 (13.3)	532 (53.6)
Preschool	0 (0.0)	25 (10.6)	112 (24.4)	137 (13.8)
Primary school	0 (0.0)	0 (0.0)	145 (31.6)	147 (14.8)
Age to go but doesn't go	0 (0.0)	21(8.9)	124 (27.0)	145 (14.6)
Missed information	7 (2.3)	9 (3.8)	17 (3.7)	31 (3.1)
Bednet information **				
Reported to have a bednet	261 (87.9)	197 (83.5)	383 (83.4)	841 (84.8)
Slept under a bednet last night	261 (87.9)	196 (83.1)	373 (81.3)	830 (83.7)
used treated bednet (LLIN)	259 (87.2)	191 (80.9)	370 (80.6)	820 (82.7)
Bednet with holes	69 (23.2)	54 (22.9)	84 (18.3)	207 (20.9)
Reported dewormed past 6 months **				
Albendazole	90 (30.3)	116 (49.2)	205 (44.7)	411 (41.4)
Mebendazole	43 (14.5)	42 (17.8)	103 (22.4)	188 (18.9)
Don’t know	2 (0.7)	24 (10.2)	29 (6.3)	55 (5.5)

Note: Data are number (%) of participants or infection, unless otherwise indicated.

LLIN = Long Lasting Insecticide impregnated Net.

**The column total doesn’t add up to the specified total age group as the information was collected dependently.

### Distribution of infections

**Fig 2:**
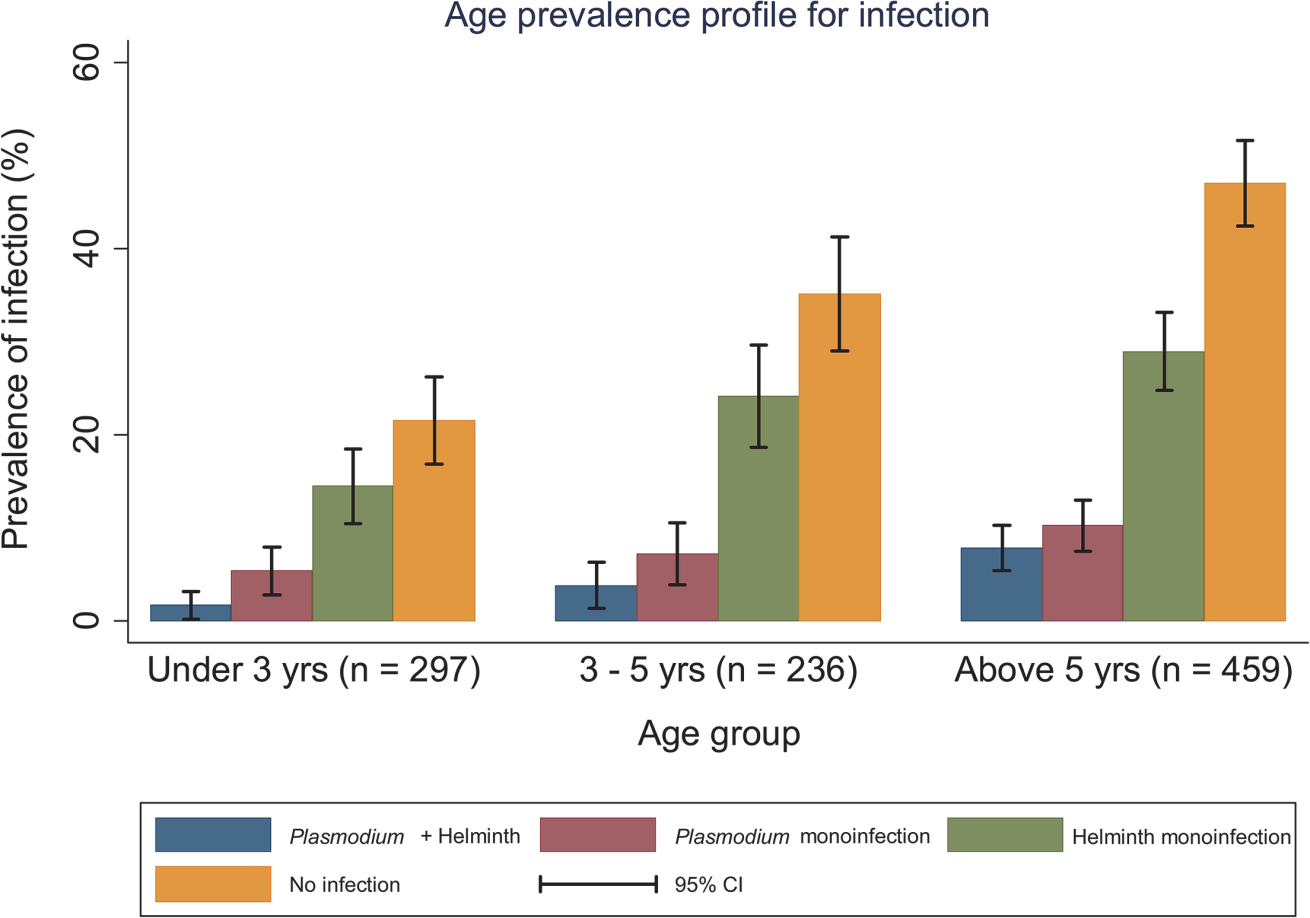
Age prevalence profile for infection (*Plasmodium* and helminth monoinfections and co-infections) within each age group.

**Fig 3:**
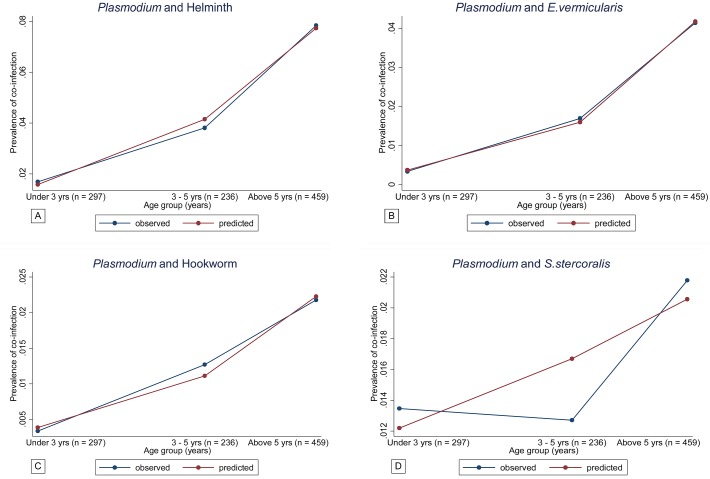
A–D Age prevalence profile of co-infection as predicted from a logistic regression model (Predicted Vs Observed prevalence). Fig 3A shows *Plasmodium* and helminth co-infection; 3B *Plasmodium* and *E*. *vermicularis* co-infection; 3C *Plasmodium* and hookworm co-infection; 3D *Plasmodium* and *S*. *stercoralis* co-infection

Prevalence of *Plasmodium*, helminth and co-infections are shown in Fig 1 and Table 3. Out of the 992 children included in the analysis, 130 (13.1%) were infected with *Plasmodium* species, 283 (28.5%) had helminth infection and 50 (5%) harbored both infections (co-infected). The prevalence of *Plasmodium* and helminth monoinfection were 8.1% (80/992) and 23.5% (233/992) respectively. *E*. *vermicularis* was the most prevalent single helminth infection 116 (11.7%) followed by hookworm 60 (6.1%) and *S*. *stercoralis* 42 (4.2%). The prevalence of *Plasmodium*, STH and co-infections increased with age, older children were mostly affected (Fig 2 and 3A and Table 3). This was especially true for the most prevalent infections, namely *E*. *vermicuralis* and hookworm infections, co-infected or not with *Plasmodium (* Fig 3B and 3C). The only exception was the prevalence of *S*. *stercoralis* monoinfection which was slightly higher in children below five years of age (Table 3 and Fig 3D). Co-infection with *Plasmodium* and *S*. *hematobium* was found in two children (0.2%) and co-infection with *Plasmodium* and *T*. *trichura* in only one child (0.1%), all above five years of age. Two children (0.2%) had positive ICT for *W*. *bancrofti* infection.

**Table 3:** Prevalence of *Plasmodium* and helminth infections of study participants by age group.

Characteristics	< 3 years (n = 297)	3–5 years (n = 236)	> 5 years (n = 459)	Total (%) N = 992
**All *Plasmodium* infection**				
*Plasmodium* (+ve)	21 (7.1)	26 (11.0)	83 (18.1)	130 (13.1)
*Plasmodium* (-ve)	276 (92.9)	210 (89.0)	376 (81.9)	862 (86.9)
**Geometric mean parasite count** (25^th^–75^th^ Quartile)	1993 (1200–6740)	1896 (1260–2680)	979 (480–1600)	1227 (560–2200)
*Plasmodium* monoinfection	16 (5.4)	17 (7.2)	47 (10.2)	80 (8.1)
**All helminth infection**				
Helminth (+ve)	48 (16.2)	66 (28.0)	169 (36.8)	283 (28.5)
Helminth (-ve)	249 (83.8)	170 (72.0)	290 (63.2)	709 (71.5)
**Single helminth infection**				
All single infection	41 (13.8)	52 (22.0)	140 (30.5)	233 (23.5)
*E*. *vermicularis*	17 (5.7)	25 (10.6)	74 (16.1)	116 (11.7)
Hookworm	8 (2.7)	14 (5.9)	38 (8.3)	60 (6.1)
*S*. *stercoralis*	13 (4.4)	11 (4.7)	18 (3.9)	42 (4.2)
*T*. *trichura*	2 (0.7)	2 (0.8)	7 (1.5)	11 (1.1)
*S*. *haematobium*	0 (0.0)	0 (0.0)	2 (0.4)	2 (0.2)
*W*. *bancrofti*	1 (0.3)	0 (0.0)	1 (0.2)	2 (0.2)
**Mixed helminth infection**				
Double helminth species	7 (2.4)	12 (5.1)	24 (5.2)	43 (4.3)
> 2 helminth species	0 (0.0)	2 (0.8)	5 (1.1)	7 (0.7)
***Plasmodium* and helminth co-infection**				
All *Plasmodium* + helminth co-infection	5 (1.7)	9 (3.8)	36 (7.8)	50 (5.0)***
*Plasmodium* + *E*. *vermicularis*	1 (0.3)	4 (1.7)	19 (4.1)	24 (2.4)
*Plasmodium* + hookworm	1 (0.3)	3 (1.3)	10 (2.2)	14 (1.4)
*Plasmodium* + *S*. *stercoralis*	4 (1.4)	3 (1.3)	10 (2.2)	17 (1.7)
*Plasmodium* + *T*. *trichura*	0 (0.0)	0 (0.0)	1 (0.2)	1 (0.1)
*Plasmodium* + *S*. *hematobium*	0 (0.0)	0 (0.0)	2 (0.4)	2 (0.2)

*** The total below is more than 5.0% as some specific *Plasmodium* helminth co-infection have more than one helminth species.

**Fig 4:**
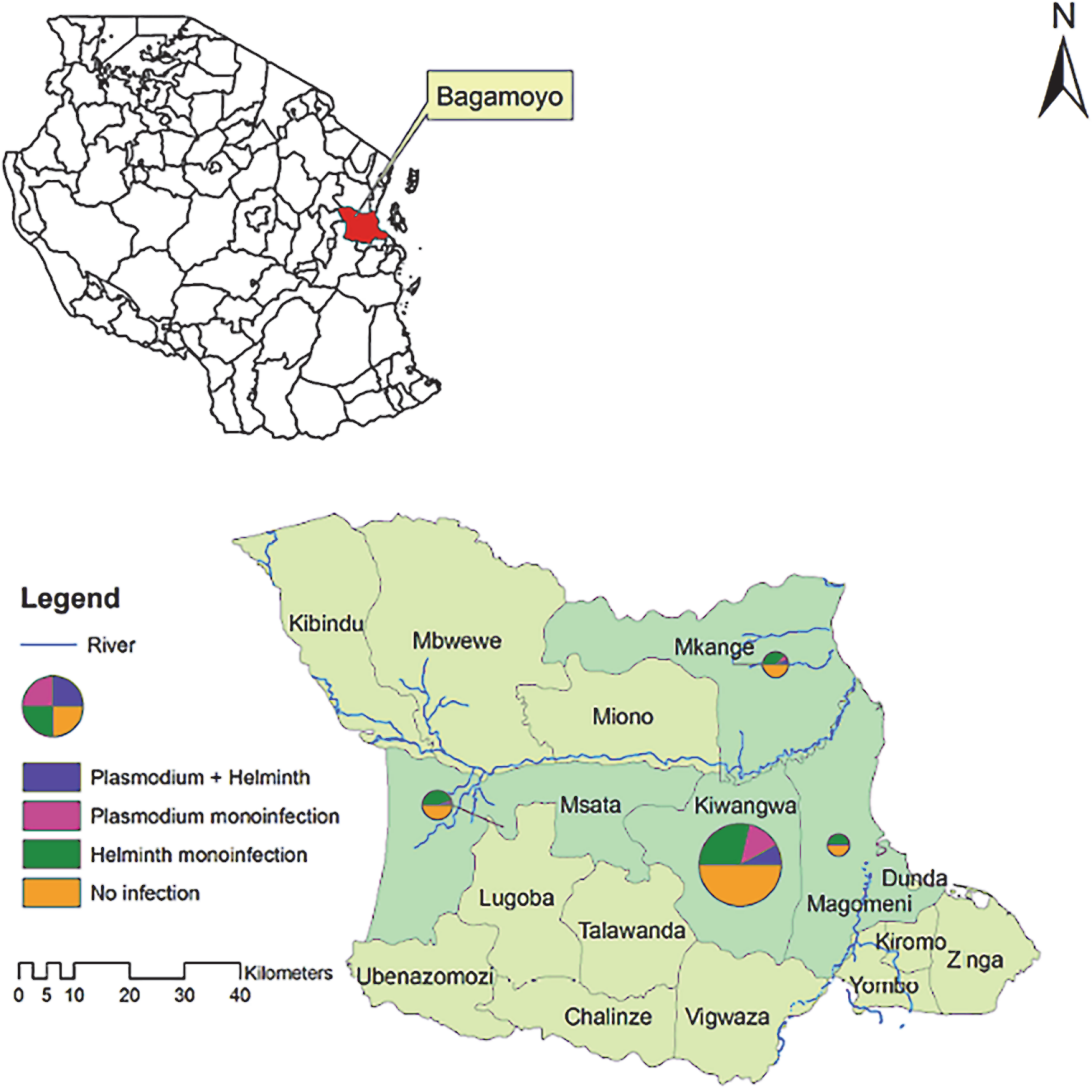
Administrative map of Bagamoyo district, coastal region of Tanzania and the spatial distribution of monoinfection and co-infections within four villages, namely Magomeni, Kiwangwa, Msata and Mkange. The size of the pie is proportional to the sample size contributed by each village/hamlet.

**Fig 5:**
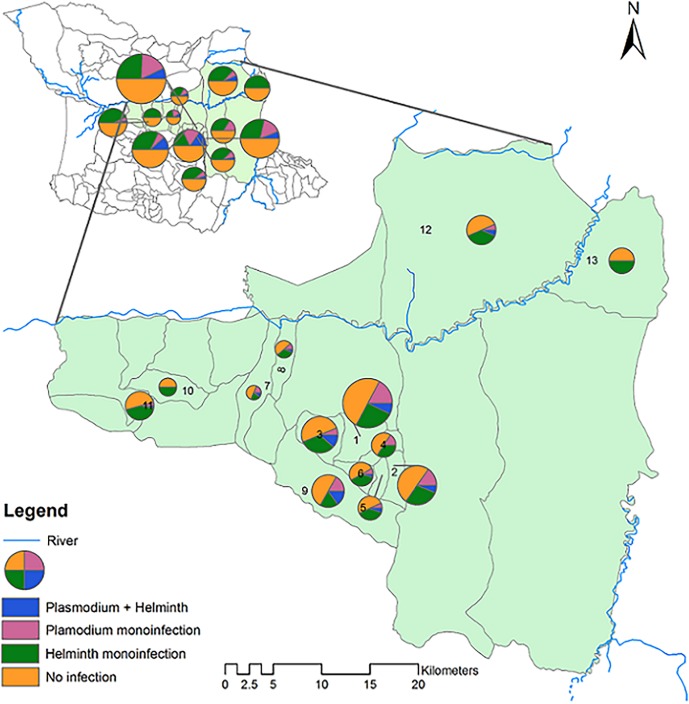
Spatial distribution of monoinfection and co-infection status among hamlets of the four villages within Bagamoyo, coastal region of Tanzania. The size of the pie is proportional to the sample size contributed by each village/hamlet. 1 = Kiwangwa kiwangwa 2 = Kiwangwa Mwavi 3 = Kiwangwa Bago 4 = Kiwangwa Kibaoni 5 = Kiwangwa Kwambwela 6 = Kiwangwa Pipani 7 = Kiwangwa Masuguru 8 = Kiwangwa Mwetemo 9 = Kiwangwa Msinune 10 = Msata Kihangaiko 11 = Msata Msata 12 = Mkange Matipwili 13 = Magomeni (Makurunge—Kitame).

Fig 4 shows administrative map of Tanzania locating Bagamoyo district within coastal region and the spatial distribution of monoinfection/co-infections prevalence in the four villages studied namely Kiwangwa, Mkange, Msata and Magomeni. Spatial heterogeneity of infection prevalence was observed between and within villages. Fig 5 shows the distribution of monoinfection and co-infections among the hamlets of the four studied villages. There were significantly different prevalences of helminth ranging from 44.7% in Mkange to 26.3% in Kiwangwa. The prevalence of *Plasmodium* infection ranged from 15.4% in Kiwangwa to zero in Magomeni village, with co-infection prevalence being higher in the hamlet of Kiwangwa, Kiwangwa Msinune (p = 0.028), Kiwangwa Bago and Mkange Matipwili (Table 4 and Fig 4 and 5).

**Table 4:** Variables associated with *Plasmodium*, STH and *Plasmodium* + STH co-infection using bivariate analysis.

Risk factors	*Plasmodium* infection		STH infection		*Plasmodium* + STH co-infection	
	OR (95% CI)	p-value	OR (95% CI)	p-value	OR	p-value
**Gender (Female (Ref))**						
Male sex	0.9 (0.6–1.2)	0.423	0.9 (0.7–1.2)	0.476	0.9 (0.6–1.7)	0.9767
**Age**						
Age in years	1.2 (1.1–1.3)	< 0.001	1.2 (1.1–1.3)	<0.001	1.2 (1.1–1.4)	0.0001
**Age group** (<3 years (Ref))						
3–5 years	1.6 (0.9–3.0)	0.113	2.0 (1.3–3.1)	0.001	2.3 (0.8–7.0)	0.137
> 5years	2.9 (1.7–4.8)	< 0.001	3.0 (2.1–4.3)	<0.001	5.0 (1.9–12.8)	0.001
**Education level** (Too young (Ref))						
Preschool	0.9 (0.5–1.8)	0.868	1.6 (1.1–2.4)	0.024	1.4 (0.6–3.5)	0.409
Primary	2.0 (1.2–3.4)	0.006	1.8 (1.2–2.6)	0.004	2.0 (0.9–4.3)	0.092
Age to go but doesn't	3.0 (1.9–4.8)	< 0.001	1.8 (1.2–2.6)	0.005	2.9 (1.4–5.9)	0.004
**Villages (Hamlets),** (Kiwangwa Kiwangwa (Ref))						
Kiwangwa Mwavi	1.0 (0.5–1.8)	0.99	1.3 (0.8–2.1)	0.358	1.0 (0.3–2.7)	0.959
Kiwangwa Bago	0.9 (0.5–1.7)	0.77	2.1 (1.3–3.4)	0.004	2.1 (0.9–5.1)	0.094
Kiwangwa Kibaoni	0.5 (0.2–1.5)	0.248	1.0 (0.5–2.0)	0.931	Omitted	-
Kiwangwa Kwambwela	0.6 (0.2–1.6)	0.285	2.2 (1.1–4.2)	0.019	0.8 (0.2–4.0)	0.826
Kiwangwa Pipani	0.3 (0.1–1.2)	0.085	0.9 (0.4–2.0)	0.839	0.4 (0.1–3.4)	0.414
Kiwangwa (Mwetemo + Masuguru)	1.3 (0.6–2.9)	0.526	1.6 (0.8–3.3)	0.183	1.4 (0.4–5.4)	0.593
Kiwangwa Msinune	1.7 (0.9–3.1)	0.07	1.3 (0.7–2.2)	0.413	2.7 (1.1–6.7)	0.028
Msata (Msata + Kihangaiko)	0.2 (0.1–0.6)	0.004	1.4 (0.8–2.4)	0.244	0.2 (0.0–1.7)	0.14
Mkange Matipwili	0.8 (0.4–1.7)	0.589	3.0 (1.7–5.2)	< 0.001	1.4 (0.5–4.4)	0.51
Magomeni (Makurunge—Kitame)	Omitted	-	2.3 (1.2–4.3)	0.008	Omitted	-
**Did not sleep under bednet last night** (Ref)						
Slept under bednet last night	0.5 (0.3–0.9)	0.0193	0.7 (0.4–1.2)	0.1951	1.2 (0.4–4.1)	0.7206
**Did not use antihelminth past 6 months** (Ref)	** **	** **	** **	** **	** **	** **
Albendazole	1.0 (0.7–1.5)	0.8397	1.5 (1.1–2.0)	0.004	1.7 (0.9–3.0)	0.0679
Mebendazole	0.9 (0.5–1.4)	0.6066	1.3 (0.9–1.8)	0.1617	0.7 (0.3–1.5)	0.3444
**Helminth negative (Ref)**						
Any helminth	1.7 (1.1–2.5)	0.0072				
*E*. *vermicularis* monoinfection	0.8 (0.4–1.5)	0.43				
Hookworm monoinfection	0.8 (0.4–1.7)	0.5423				
*S*. *stercoralis* monoifection	2.5 (1.2–5.2)	0.0146				
*T*. *trichuris* monoinfection	-	0.1177				
***Plasmodium* density**			1.0 (0.9–1.0)	0.6971	1.0 (1.0–1.0)	<0.001
**Low density parasitemia**	** **		0.5 (0.1–2.2)	0.3844	3.1 (0.6–16.0)	0.147

Note: Density of parasitemia was defined as: Low density parasite <5000/μL and high density ≥5000/ μL

CI, confidence interval; OR, odds ratio

### Relationship between *Plasmodium* and STH infections and predictors of co-infections

There was a pattern for *Plasmodium* to be associated with helminth infection [OR = 1.7, 95% CI (1.1–2.5)], which was marked for *S*. *stercoralis* monoinfection [OR = 2.5, 95% CI (1.2–5.2), p = 0.0146] as shown in Table 4. The effect was statistically significant in a multivariate negative binomial regression model when other factors were considered [IRR = 2.0, 95% CI (1.0–4.00), p = 0.034] as shown in Table 5.

**Table 5:** Association between *Plasmodium* and STH infection by multivariate analysis (Negative binomial regression).

Independent variables	All helminth^a^	*E*. *vermicularis* ^*b*^	Hookworm^c^	*S*. *stercoralis* ^*d*^	*T*. *trichura* ^*e*^
	Adjusted IRR (95% CI)	Adjusted IRR (95% CI)	Adjusted IRR (95% CI)	Adjusted IRR (95% CI)	Adjusted IRR (95% CI)
	1.3 (0.9–1.9)	1.0 (0.5–1.8)	0.6 (0.3–1.2)	1.8 (1.0–3.4)*	0.3 (0.0–1.9)
**Age group** ^**f**^					
< 3 years (Ref)					
3–5 years	1.8 (0.9–3.4)	2.3 (0.6–8.8)	2.4 (0.6–9.2)	2.5 (0.6–9.5)	2.5 (0.6–9.3)
> 5 years	3.2 (1.6–6.6)*	4.3 (1.1–16.9)*	4.5 (1.1–17.9) *	4.7 (1.2–18.8)*	4.5 (1.0–17.9)*
**Education level** ^g^					
Too young (Ref)					
Preschool	0.4 (0.2–0.9)*	0.5 (0.2–1.6)	0.5 (0.2–1.5)	0.5 (0.2–1.5)	0.5 (0.2–1.5)
Primary	0.9 (0.5–1.7)	0.6 (0.2–1.7)	0.6 (0.2–1.6)	0.6 (0.2–1.7)	0.6 (0.2–1.7)
Age to go but doesn’t	1.3 (0.7–2.3)	1.1 (0.4–2.9)	1.1 (0.4–2.8)	1.2 (0.4–3.1)	1.0 (0.4–2.8)
Village (Hamlets) ^h^	0.9 (0.9–1.0)	0.9 (0.9–1.0)	0.9 (0.9–1.0)	1.0 (0.9–1.0)	1.0 (0.9–1.1)
Slept under bednet last night ^i^	0.7 (0.4–1.3)	2.1 (0.6–7.0)	1.9 (0.6–6.5)	2.1 (0.6–6.8)	2.0 (0.6–6.7)
**Use of antihelminth past 6 months** ^j^					
Albendazole	1.0 (0.8–1.3)	1.0 (0.5–2.0)	1.0 (0.5–2.0)	1.0 (0.5–2.0)	0.9 (0.5–1.8)
Mebendazole	0.8 (0.6–1.1)	0.5 (0.2–1.3)	0.4 (0.2–1.2)	0.5 (0.2–1.3)	0.5 (0.2–1.2)

IRR is the incidence rate ratio

All the models a, b, c, d and e were adjusted for f, g, h, i and j.

^a^Reference group was helminth negative

^b, c, d, e^ Reference group were other worm positive species; Variable village (Hamlets)—Report on hamlets categorization was removed as were insignificant when the model was run.

*p values were significant

The risk of *Plasmodium*, STH and *Plasmodium* STH co-infections increased with age, with older children (>5 years) being more affected compared to younger children (<3 years and 3–5 years). The differences were statistically significant by bivariate and multivariate negative binomial regression analysis (Tables 4 and 5). Overall there was an age pattern for *Plasmodium* to be associated with STH [multivariate negative binomial regression [IRR = 2.9, 95% CI (1.7–5.1)], which was more marked for *S*. *stercoralis* [IRR = 3.9, 95% CI (1.2–13.1)], and especially in young children. Mantel—Haenszel stratified ORs (Table 6) indicated that the risk of *Plasmodium* infection with *S*. *stercoralis* was higher among younger children aged below 3 years (stratum specific OR = 9.2, 95% CI (0.8–105.5) when exploring for confounding effect of age groups (M-H adjusted OR = 2.2, 95% CI (1.1–4.3), p = 0.0266; homogeneity of ORs, p = 0.3774). Compared to the results with other type of STH, *S*. *stercoralis* infection showed increased risk of *Plasmodium* among younger age group but the homogeneity test suggests no difference in the odds between age groups.

**Table 6:** Association between *Plasmodium* and STH infection by Mantel-Haenszel analysis using age group as justification.

Age group (years)	All helminth	*E*. *Vermicularis*	Hookworm	*S*. *stercoralis*	*T*. *trichura*
	OR (95% CI)	OR (95% CI)	OR (95% CI)	OR (95% CI)	OR (95% CI)
Under 3 years	1.7 (0.6–4.9)	0.3 (0.0–3.2)	0.5 (0.0–5.2)	9.2 (0.8–105.5)	0.0
	p = 0.3241	p = 0.3042	p = 0.5704	p = 0.0293	p = 0.6259
3–5 years	1.4 (0.6–3.4)	0.8 (0.2–3.2)	0.9 (0.2–3.8)	1.5 (0.3–7.1)	0.0
	p = 0.4242	p = 0.7219	p = 0.8401	p = 0.5789	p = 0.2698
Above 5 years	1.4 (0.9–2.3)	0.9 (0.4–1.8)	0.7 (0.3–1.5)	1.8 (0.8–4.4)	0.3 (0.0–2.1)
	p = 0.1718	p = 0.6995	p = 0.3129	p = 0.1604	p = 0.178
**Mixed helminth infection**					
Crude OR (95% CI)	1.7 (1.1–2.5)	0.8 (0.4–1.5)	0.7 (0.3–1.3)	1.9 (1.0–3.7)#	0.2 (0.0–1.5)
	p = 0.0072	p = 0.5392	p = 0.2784	p = 0.0588	p = 0.0811
**Helminth monoinfection**					
Crude OR (95% CI)	-	0.8 (0.4–1.5)	0.8 (0.4–1.7)	2.5 (1.2–5.2) #	0.0
	-	p = 0.43	p = 0.5423	p = 0.0146	p = 0.1177
M-H adjusted for age group^a^					
OR (95% CI)	1.4 (1.0–2.1)	0.8 (0.4–1.4)	0.7 (0.3–1.3)	2.2 (1.1–4.3)	0.2 (0.0–1.5)
	p = 0.0684	p = 0.4125	p = 0.2641	p = 0.0266	p = 0.0739
Homogeneity of ORs^b^	p = 0.9491	p = 0.7055	p = 0.9272	p = 0.3774	p = 0.8257

# Denotes crude ORs. To assess for confounding, crude and adjusted ORs are compared in terms of the difference in relation to magnitude

^a^ The test assesses whether the exposure is significant after adjusting for the age groups

^b^ The test compares whether there is significant difference between age group specific ORs (homogeneity of the stratum ORs), hence whether the overall adjusted OR is valid.

In addition, children who were not attending school although they should have according to their age had increased risk of *Plasmodium* and co-infections by bivariate analysis although the statistical association was lost in a multivariate analysis. Male and female children were equally affected (Table 4).

The geometric mean *Plasmodium* parasite count decreased with age as shown in Table 3. Most of the studied children had low intensity helminth infections. Moderate and heavy STH infections intensity were noted among older children. Generally, there were no significant correlation between *Plasmodium* and STH densities. There was a trend for a negative correlation between *Plasmodium* parasite density and *S*. *stercoralis* larvae count (r = -0.0786, p = 0.8082) and a positive correlation with a hookworm (r = 0.2123, p = 0.5560) and *E*. *vermicularis* (r = 0.0418, p = 0.7095) infections.

## Discussion

The current study provides baseline epidemiological data of *Plasmodium* and STH infections in the whole population of children, including the young ones who are rarely surveyed for the latter. To increase sensitivity, different diagnostic methods for helminth infection were used including adhesive tape slides for *E*. *vermicularis* and Baermann technique for *S*. *stercoralis*. This enabled us to have full range of helminth pattern within the area where malaria transmission occurs throughout the year and National malaria and helminth control programs are undertaken.

Results show that infection with *Plasmodium*, STH and co-infections are common among children aged below five and above five years living in Bagamoyo district, Tanzania. Despite high coverage of LLINs (above 80%), pockets of *Plasmodium* infection remain in west side areas like Kiwangwa compared to Magomeni village which is closer to Bagamoyo town where malaria prevalence was documented to be low [21]. This spatial variation could be explained by behavioral factors such as outdoor activities, coverage and effective use of LLINs in the Magomeni village and easy access to health care and hence effective treatment. The geometric mean *Plasmodium* parasite count decreased with advancing age as expected in most of the malaria endemic countries in relation to development of antimalarial specific immunity [30,31] but the malaria infection prevalence increased with age. These findings may be an indication of a shift of *Plasmodium* infection towards older age group as observed in Muheza district, Tanzania [32] and other parts of Africa [33–35] where malaria transmission tends to decline and acquisition of immunity is thus delayed. Prevention strategies need thus to take into account the older age group too in the momentum of malaria elimination [36].

The association of STH and *Plasmodium* infections highlights the extent of the burden of parasites in older age groups. Our results show a definite increase of parasite prevalence, especially STH, with age, as do most of the previous studies [5,6,17,37,38]. The prevalence and pattern of co-infections observed in Bagamoyo differ from those reported in Magu [6] and Mvomero districts [17], Tanzania. Our results show lower prevalence of helminth and *Plasmodium* helminth co-infections with predominance of *E*. *vermicularis*, hookworm and *S*. *stercoralis*. The method of detection might be the reason for these differences. Indeed, we used adhesive tape slides for *E*. *vermicularis* and Baermann technique for *S*. *stercoralis* together with Kato-Katz technique, which detected these specific worms, mostly missed in other surveys. Factors such as exposure and intervention coverage may also explain the types of helminth infection isolated and high prevalence in Magu and Mvomero districts. In Tanzania, published reports on mass drug administration (MDA), mostly under National Lymphatic Filariasis Elimination Program (NLFEP), have shown coverage to fluctuate [39] and its effectiveness to vary depending on the chemoprophylaxis used and duration between cycles [40,41]. The uptake variations could results into persistence of parasites in certain areas and subgroups. In this study, an increased risk of STH, *Plasmodium* and co-infections was observed among the school-aged children who were not schooling. These may have missed the opportunity to be dewormed, either at school level or within the under-fives program. These children may also be exposed to other risk factors in the environment, or behave differently in terms of sanitation and hygiene. The latter have not been assessed in the present study, which represents a definite limitation. Such a burden in this age group is not only deleterious for them but also acts as a reservoir of infection within the population [42].

Co-infection patterns increased with age as predicted from the age specific prevalence rates by the simple probability model except for *S*. *stercoralis* co-infection. The exposure of *S*. *stercoralis and Plasmodium* co-infections was significantly different from the other species of helminth. The results show that children with *S*. *stercoralis* had twice the risk of *Plasmodium* infection, with even higher odds among children below 3 years of age, as compared to those with other species of STH. This observation requires further exploration as reports on the age profile for *S*. *stercoralis* infection are rare and still conflicting [43–46]. A study done in Côte d’Ivoire showed that hookworm and *S*. *stercoralis* had an almost parallel shape of age prevalence pattern [38], but it did not stratify the <5 children into smaller age categories. This could be the reason for the masked trend compared to what was observed in the present study where a negative correlation between *S*. *stercoralis* larvae and *Plasmodium* parasitemia was shown compared to positive correlation with hookworm and *E*. *vermicularis*, although not significant. The observed relation between *Plasmodium* and *S*. *stercoralis* infection may arise due to biological associations, whereby *S*. *stercoralis* being an early life and chronic infection, starts in the first years and persists through older aged groups promoting establishment and/or survival of *Plasmodium* infection potentially through Th2 lymphocytes immune modulation [47]. It has been repeatedly shown that hookworm tends to exaggerate *Plasmodium* infection but knowledge on the immunomodulation with *S*. *stecoralis* co-infection is still scarce. Depending on the pro and anti-inflammatory responses mounted within the host, immune response could either promote or inhibit *Plasmodium* infection [16,47,48]. The equilibrium with *S*. *stercoralis* could have been maintained through its intrinsic characteristic to persist for many years in asymptomatic immunocompetent host surviving through low grade autoinfection cycles [47]. In this study, children were not tested for HIV infection which is among the risk factor for *S*. *stercoralis* hyperinfection syndrome [49–51]. The immaturity and predominance of Th2 response among younger children could also explain the increased risk of *Plasmodium* co-infection with *S*. *stercoralis* [52].

Heterogeneity of infection prevalence within and between villages indicate that other factors apart from biological association determine the co-infection patterns. Behavioural such as outdoor activities and walking barefooted, sanitation, hygiene and socio-economic factors could explain the variations [38]. Considering the type of STH species isolated and their modes of transmission, exposure factors conducive to both parasites are suspected to be main contributors of *Plasmodium* and STH co-infections within the studied population [11]. Previous studies done within Bagamoyo district in 2004 suggested that availability of safe water is a serious problem with public health consequences [22]. Up to 40% of people reporting water to be not easily accessible [22] and 70.8% of the water sources were contaminated with fecal coliforms [23]. The situation has not much changed, at least in the rural areas, just few kilometers from the Bagamoyo town. Recent studies conducted within the area showed high rates of water contamination [53,54]. Both hookworm and *S*. *stercoralis* are transmitted via skin penetration in poorly maintained latrines and sites of promiscuous defecation. As of *E*. *vermicularis* direct transfer of eggs into mouth, inhalation and retroinfection are possible in areas of poor hygiene and scarcity of water [55,56]. All these could contribute to high reinfection rates [57] and persistence of chronic infection post treatment.

Overall, the results of this study demonstrate that both *Plasmodium* and STH exhibit marked age dependency in infection patterns. In main land Tanzania, control program against helminth has been implemented through expanded program of immunization (EPI) using mebendazole or albendazole among <5 years children and community based Global Program to Eliminate Lymphatic Filariasis (GPELF) using ivermectin plus albendazole among school-aged children and adult population. Under five years children have been targeted by interventions against malaria via antenatal and later postnatal programs where LLINs are distributed. Generally, school-aged children have been rather neglected and therefore not well covered by both control programs. In the current study, the heaviest load of helminth infection was detected among children aged above five years underlying the importance of deworming program to be focused on this age group as suggested by the WHO in order to reduce morbidity and transmission of helminth [29]. Integrated control approaches emphasizing on health education, improvement of environmental sanitation and hygiene coupled with improved housing and access to water, chemoprophylaxis [29,42] and LLINs distributions are required considering the pattern and types of infections within the area to interrupt transmission of both STH and *Plasmodium* among both the school-aged children but also the under-fives. Frequent and effective antihelminth administrations at least twice a year with a drug like ivermectin which has shown to reduce both helminth and malaria transmission could be prioritized to reduce the burden of co-infection in school-aged children [58]. Potential safety and additional impact of ivermectin to reduce malaria requires further exploration considering the risk of co-infection early in childhood with *S*. *stercoralis* [58]. The risk of *Plasmodium* with *S*. *stercoralis* infection among young children requires more investigation to better understand this singular interaction.

## Supporting Information

S1 Checklist STROBE checklist. (DOC)

S1 Dataset Dataset in STATA format with the variables used for the community survey analysis. (ZIP)

## References

[1] WHO (2013) World malaria report: 2013: World Health Organization.

[2] RBM (2012) (Roll Back Malaria) Partnership: Progress and Impact Series 3, 2012. Focus on Mainland Tanzania. Available at: http://www.rbm.who.int/ProgressImpactSeries/docs/report10-en.pdf, accessed 7 February 2014.

[3] Brooker SJ, Pullan RL, Gitonga CW, Ashton RA, Kolaczinski JH, et al (2012) *Plasmodium*-helminth coinfection and its sources of heterogeneity across East Africa. J Infect Dis 205: 841–852. 10.1093/infdis/jir844 22262792 PMC3274378

[4] Pullan RL, Kabatereine NB, Bukirwa H, Staedke SG, Brooker S (2011) Heterogeneities and consequences of *Plasmodium* species and hookworm coinfection: a population based study in Uganda. J Infect Dis 203: 406–417. 10.1093/infdis/jiq063 21187338 PMC3038339

[5] Mazigo HD, Waihenya R, Lwambo NJ, Mnyone LL, Mahande AM, et al (2010) Co-infections with Plasmodium falciparum, Schistosoma mansoni and intestinal helminths among schoolchildren in endemic areas of northwestern Tanzania. Parasit Vectors 3: 44 10.1186/1756-3305-3-44 20482866 PMC2881914

[6] Kinung'hi SM, Magnussen P, Kaatano GM, Kishamawe C, Vennervald BJ (2014) Malaria and Helminth Co-Infections in School and Preschool Children: A Cross-Sectional Study in Magu District, North-Western Tanzania. PLoS ONE 9: e86510 10.1371/journal.pone.0086510 24489732 PMC3906044

[7] Steinmann P, Utzinger J, Du Z-W, Zhou X-N (2010) Multiparasitism: a neglected reality on global, regional and local scale. Adv Parasitol 73: 21–50. 10.1016/S0065-308X(10)73002-5 20627138

[8] Petney TN, Andrews RH (1998) Multiparasite communities in animals and humans: frequency, structure and pathogenic significance. Int J Parasitol 28: 377–393. 9559357 10.1016/s0020-7519(97)00189-6

[9] Rothman KJ, Greenland S (2005) Causation and causal inference in epidemiology. Am J Public Health 95 Suppl 1: S144–150. 16030331 10.2105/AJPH.2004.059204

[10] Brooker S, Clements AC (2009) Spatial heterogeneity of parasite co-infection: Determinants and geostatistical prediction at regional scales. Int J Parasitol 39: 591–597. 10.1016/j.ijpara.2008.10.014 19073189 PMC2644303

[11] Booth M (2006) The role of residential location in apparent helminth and malaria associations. Trends in parasitology 22: 359–362. 16797235 10.1016/j.pt.2006.06.007

[12] Brooker S, Akhwale W, Pullan R, Estambale B, Clarke SE, et al (2007) Epidemiology of plasmodium-helminth co-infection in Africa: populations at risk, potential impact on anemia, and prospects for combining control. Am J Trop Med Hyg 77: 88–98. 18165479 PMC2637949

[13] Druilhe P, Tall A, Sokhna C (2005) Worms can worsen malaria: towards a new means to roll back malaria? Trends Parasitol 21: 359–362. 15967721 10.1016/j.pt.2005.06.011

[14] Nacher M, Singhasivanon P, Yimsamran S, Manibunyong W, Thanyavanich N, et al (2002) Intestinal helminth infections are associated with increased incidence of *Plasmodium falciparum* malaria in Thailand. Journal of Parasitology 88: 55–58. 12053980 10.1645/0022-3395(2002)088[0055:IHIAAW]2.0.CO;2

[15] Spiegel A, Tall A, Raphenon G, Trape J-F, Druilhe P (2003) Increased frequency of malaria attacks in subjects co-infected by intestinal worms and *Plasmodium falciparum* malaria. Transactions of the Royal Society of Tropical Medicine and Hygiene 97: 198–199. 14584377 10.1016/s0035-9203(03)90117-9

[16] Nacher M (2011) Interactions between worms and malaria: Good worms or bad worms? Malar J 10: 259 10.1186/1475-2875-10-259 21910854 PMC3192711

[17] Mboera LE, Senkoro KP, Rumisha SF, Mayala BK, Shayo EH, et al (2011) *Plasmodium falciparum* and helminth coinfections among schoolchildren in relation to agro-ecosystems in Mvomero District, Tanzania. Acta Trop 120: 95–102. 10.1016/j.actatropica.2011.06.007 21741929

[18] Mazigo HD, Ambrose-Mazigo EE (2012) Mono-parasite infection versus co-infections in Tanzania: the need to revise our research focus. Tanzania Journal of Health Research 14. 10.4314/thrb.v14i1.1 26591739

[19] Vandenbroucke JP, von Elm E, Altman DG, Gøtzsche PC, Mulrow CD, et al (2007) Strengthening the Reporting of Observational Studies in Epidemiology (STROBE): Explanation and Elaboration. PLoS Med 4: e297 17941715 10.1371/journal.pmed.0040297 PMC2020496

[20] National Bureau of Statistics, NBS (2012). Ministry of Planning, Economy and Empowerment, The United Republic of Tanzania Population and Housing Census 2012. www.nbs.go.tz, accessed 18 November 2014.

[21] Williams J, Dillip A, Smithson P, Hildon Z (CSS report-ihi, 2013) Comparing changes in morbidity and mortality in under-five year olds in Kilombero and Bagamoyo district hospitals.

[22] Kusiluka L, Mlozi M, Munishi P, Karimuribo E, Luoga E, et al (2004) Preliminary observations on accessibility and utilisation of water in selected villages in Dodoma Rural and Bagamoyo Districts, Tanzania. Physics and Chemistry of the Earth, Parts A/B/C 29: 1275–1280.

[23] Kusiluka L, Karimuribo E, Mdegela R, Luoga E, Munishi P, et al (2005) Prevalence and impact of water-borne zoonotic pathogens in water, cattle and humans in selected villages in Dodoma Rural and Bagamoyo districts, Tanzania. Physics and Chemistry of the Earth, Parts A/B/C 30: 818–825.

[24] Willyard C (2009) Large trial to examine parasites' influence on global killers. Nat Med 15: 1097–1097. 10.1038/nm1009-1097 19812549

[25] Tanzania HIV/AIDS and Malaria Indicator Survey (2012) ICF International Calverton, Maryland USA, 2011–2012

[26] Salim N, Schindler T, Abdul U, Rothen J, Genton B, et al (2014) Enterobiasis and strongyloidiasis and associated co-infections and morbidity markers in infants, preschool-and school-aged children from rural coastal Tanzania: a cross-sectional study. BMC Infect Dis 14: 644 25486986 10.1186/s12879-014-0644-7 PMC4271451

[27] WHO (2009) Malaria microscopy quality assurance manual: World Health Organization.

[28] Knopp S, Salim N, Schindler T, Karagiannis Voules DA, Rothen J, et al. (2014) Diagnostic Accuracy of Kato–Katz, FLOTAC, Baermann, and PCR Methods for the Detection of Light-Intensity Hookworm and *Strongyloides stercoralis* Infections in Tanzania. The American Journal of Tropical Medicine and Hygiene. 10.4269/ajtmh.13-0268 PMC3945701 24445211

[29] WHO (2011) Helminth control in school age children: a guide for managers of control programmes Second edition Geneva, Switzerland: World Health Organization.

[30] Doolan DL, Dobano C, Baird JK (2009) Acquired immunity to malaria. Clin Microbiol Rev 22: 13–36, Table of Contents. 10.1128/CMR.00025-08 19136431 PMC2620631

[31] Woolhouse M (1998) Patterns in parasite epidemiology: the peak shift. Parasitology today 14: 428–434. 17040835 10.1016/s0169-4758(98)01318-0

[32] Winskill P, Rowland M, Mtove G, Malima RC, Kirby MJ (2011) Malaria risk factors in north-east Tanzania. Malar J 10: 98 10.1186/1475-2875-10-98 21507217 PMC3094229

[33] O'Meara WP, Mwangi TW, Williams TN, McKenzie FE, Snow RW, et al (2008) Relationship between exposure, clinical malaria, and age in an area of changing transmission intensity. Am J Trop Med Hyg 79: 185–191. 18689622 PMC2547116

[34] Carneiro I, Roca-Feltrer A, Griffin JT, Smith L, Tanner M, et al (2010) Age-patterns of malaria vary with severity, transmission intensity and seasonality in sub-Saharan Africa: a systematic review and pooled analysis. PLoS One 5: e8988 10.1371/journal.pone.0008988 20126547 PMC2813874

[35] Mawili-Mboumba DP, Akotet MKB, Kendjo E, Nzamba J, Medang MO, et al (2013) Increase in malaria prevalence and age of at risk population in different areas of Gabon. Malaria journal 12. 10.1186/1475-2875-12-3 PMC3549767 23282198

[36] Nankabirwa J, Brooker SJ, Clarke SE, Fernando D, Gitonga CW, et al (2014) Malaria in school-age children in Africa: an increasingly important challenge. Tropical Medicine & International Health 19: 1294–1309. 25145389 10.1111/tmi.12374 PMC4285305

[37] Brooker S, Akhwale W, Pullan R, Estambale B, Clarke SE, et al (2007) Epidemiology of *Plasmodium*-helminth co-infection in Africa: populations at risk, potential impact on anemia and prospects for combining control. The American journal of tropical medicine and hygiene 77: 88 18165479 PMC2637949

[38] Becker SL, Sieto B, Silué KD, Adjossan L, Koné S, et al (2011) Diagnosis, Clinical Features, and Self-Reported Morbidity of *Strongyloides stercoralis* and Hookworm Infection in a Co-Endemic Setting. PLoS Negl Trop Dis 5: e1292 10.1371/journal.pntd.0001292 21886853 PMC3160297

[39] Parker M, Allen T (2013) Will mass drug administration eliminate lymphatic filariasis? Evidence from northern coastal Tanzania. J Biosoc Sci 45: 517–543. 10.1017/S0021932012000466 23014581 PMC3666211

[40] Simonsen PE, Derua YA, Kisinza WN, Magesa SM, Malecela MN, et al (2013) Lymphatic filariasis control in Tanzania: effect of six rounds of mass drug administration with ivermectin and albendazole on infection and transmission. BMC infectious diseases 13: 335 10.1186/1471-2334-13-335 23870103 PMC3723586

[41] Simonsen PE, Pedersen EM, Rwegoshora RT, Malecela MN, Derua YA, et al (2010) Lymphatic filariasis control in Tanzania: effect of repeated mass drug administration with ivermectin and albendazole on infection and transmission. PLoS neglected tropical diseases 4: e696 10.1371/journal.pntd.0000696 20532226 PMC2879369

[42] Campbell SJ, Savage GB, Gray DJ, Atkinson J-AM, Soares Magalhães RJ, et al (2014) Water, Sanitation, and Hygiene (WASH): A Critical Component for Sustainable Soil-Transmitted Helminth and Schistosomiasis Control. PLoS Negl Trop Dis 8: e2651 10.1371/journal.pntd.0002651 24722335 PMC3983087

[43] Steinmann P, Zhou X-N, Du Z-W, Jiang J-Y, Wang L-B, et al (2007) Occurrence of *Strongyloides stercoralis* in Yunnan Province, China, and comparison of diagnostic methods. PLoS Negl Trop Dis 1: e75 17989788 10.1371/journal.pntd.0000075 PMC2041812

[44] Egido J, De Diego J, Penin P (2001) The prevalence of enteropathy due to strongyloidiasis in Puerto Maldonado (Peruvian Amazon). Brazilian Journal of Infectious Diseases 5: 119–123. 11506774 10.1590/s1413-86702001000300003

[45] Lindo JF, Robinson RD, Terry SI, Vogel P, Gam AA, et al (1995) Age-prevalence and household clustering of *Strongyloides stercoralis* infection in Jamaica. Parasitology 110: 97–102. 7845718 10.1017/s0031182000081099

[46] Glinz D, N'Guessan NA, Utzinger J, N'Goran EK (2010) High prevalence of *Strongyloides stercoralis* among school children in rural Côte d'Ivoire. Journal of Parasitology 96: 431–433. 10.1645/GE-2294.1 19916629

[47] Concha R, Harrington WJ, Rogers AI (2005) Intestinal Strongyloidiasis: Recognition, Management, and Determinants of Outcome. Journal of Clinical Gastroenterology 39: 203–211. 15718861 10.1097/01.mcg.0000152779.68900.33

[48] Knowles SC (2011) The effect of helminth co-infection on malaria in mice: a meta-analysis. International journal for parasitology 41: 1041–1051. 10.1016/j.ijpara.2011.05.009 21777589

[49] Keiser PB, Nutman TB (2004) Strongyloides stercoralis in the Immunocompromised Population. Clin Microbiol Rev 17: 208–217. 14726461 10.1128/CMR.17.1.208-217.2004 PMC321465

[50] Marcos LA, Terashima A, DuPont HL, Gotuzzo E (2008) Strongyloides hyperinfection syndrome: an emerging global infectious disease. Transactions of the Royal Society of Tropical Medicine and Hygiene 102: 314–318. 10.1016/j.trstmh.2008.01.020 18321548

[51] Olsen A, van Lieshout L, Marti H, Polderman T, Polman K, et al (2009) Strongyloidiasis–the most neglected of the neglected tropical diseases? Transactions of the Royal Society of Tropical Medicine and Hygiene 103: 967–972. 10.1016/j.trstmh.2009.02.013 19328508

[52] PrabhuDas M, Adkins B, Gans H, King C, Levy O, et al (2011) Challenges in infant immunity: implications for responses to infection and vaccines. Nature immunology 12: 189 10.1038/ni0311-189 21321588

[53] Mattioli MC, Boehm AB, Davis J, Harris AR, Mrisho M, et al (2014) Enteric Pathogens in Stored Drinking Water and on Caregiver’s Hands in Tanzanian Households with and without Reported Cases of Child Diarrhea. PloS one 9: e84939 10.1371/journal.pone.0084939 24392161 PMC3879350

[54] Mattioli MC, Pickering AJ, Gilsdorf RJ, Davis J, Boehm AB (2012) Hands and water as vectors of diarrheal pathogens in Bagamoyo, Tanzania. Environmental science & technology 47: 355–363. 10.1021/es303878d 23181394

[55] Knight R (1982) Parasitic disease in man: Edinburgh, UK; Churchill Livingstone.

[56] Cook G (1994) *Enterobius vermicularis* infection. Gut 35: 1159–1162. 7959218 10.1136/gut.35.9.1159 PMC1375686

[57] Brooker S, Bethony J, Hotez PJ (2004) Human hookworm infection in the 21st century. Adv Parasitol 58: 197–288. 15603764 10.1016/S0065-308X(04)58004-1 PMC2268732

[58] Slater HC, Walker PG, Bousema T, Okell LC, Ghani AC (2014) The potential impact of adding ivermectin to a mass treatment intervention to reduce malaria transmission: a modelling study. J Infect Dis 210: 1972–1980. 10.1093/infdis/jiu351 24951826

