# Supplementary material for: Distribution and Risk Factors for Plasmodium and Helminth Co-infections: A Cross-Sectional Survey among Children in Bagamoyo District, Coastal Region of Tanzania
Source: PLoS Negl Trop Dis. 2015 Apr 2;9(4):e0003660. doi: 10.1371/journal.pntd.0003660 (PMC4383605; doi:10.1371/journal.pntd.0003660)
Supplement: S1 Checklist — (DOC) [file pntd.0003660.s001.doc]

STROBE Statement—checklist of items that were applicable in reporting of the current cross sectional study

|  | Item No | Recommendation |
| --- | --- | --- |
| **Title and abstract** | 1 | 🗹 (*a*) Indicate the study’s design with a commonly used term in the title or the abstract |
|  |  | 🗹 (*b*) Provide in the abstract an informative and balanced summary of what was done and what was found |
| Introduction | | |
| Background/rationale | 2 | 🗹 Explain the scientific background and rationale for the investigation being reported |
| Objectives | 3 | 🗹 State specific objectives, including any prespecified hypotheses |
| Methods | | |
| Study design | 4 | 🗹 Present key elements of study design early in the paper |
| Setting | 5 | 🗹 Describe the setting, locations, and relevant dates, including periods of recruitment, exposure, follow-up, and data collection |
| Participants | 6 | (*a*) *Cohort study*—Give the eligibility criteria, and the sources and methods of selection of participants. Describe methods of follow-up  *Case-control study*—Give the eligibility criteria, and the sources and methods of case ascertainment and control selection. Give the rationale for the choice of cases and controls  🗹 *Cross-sectional study*—Give the eligibility criteria, and the sources and methods of selection of participants |
|  |  | (*b*) *Cohort study*—For matched studies, give matching criteria and number of exposed and unexposed  *Case-control study*—For matched studies, give matching criteria and the number of controls per case |
| Variables | 7 | 🗹 Clearly define all outcomes, exposures, predictors, potential confounders, and effect modifiers. Give diagnostic criteria, if applicable |
| Data sources/ measurement | 8* | 🗹 For each variable of interest, give sources of data and details of methods of assessment (measurement). Describe comparability of assessment methods if there is more than one group |
| Bias | 9 | Describe any efforts to address potential sources of bias |
| Study size | 10 | 🗹 Explain how the study size was arrived at |
| Quantitative variables | 11 | Explain how quantitative variables were handled in the analyses. If applicable, describe which groupings were chosen and why |
| Statistical methods | 12 | 🗹 (*a*) Describe all statistical methods, including those used to control for confounding |
|  |  | (*b*) Describe any methods used to examine subgroups and interactions |
|  |  | (*c*) Explain how missing data were addressed |
|  |  | (*d*) *Cohort study*—If applicable, explain how loss to follow-up was addressed  *Case-control study*—If applicable, explain how matching of cases and controls was addressed  *Cross-sectional study*—If applicable, describe analytical methods taking account of sampling strategy |
|  |  | (*e*) Describe any sensitivity analyses |

| Results | | |
| --- | --- | --- |
| Participants | 13* | 🗹 (a) Report numbers of individuals at each stage of study—eg numbers potentially eligible, examined for eligibility, confirmed eligible, included in the study, completing follow-up, and analysed |
|  |  | (b) Give reasons for non-participation at each stage |
|  |  | 🗹 (c) Consider use of a flow diagram |
| Descriptive data | 14* | 🗹 (a) Give characteristics of study participants (eg demographic, clinical, social) and information on exposures and potential confounders |
|  |  | 🗹 (b) Indicate number of participants with missing data for each variable of interest |
|  |  | (c) *Cohort study*—Summarise follow-up time (eg, average and total amount) |
| Outcome data | 15* | *Cohort study*—Report numbers of outcome events or summary measures over time |
|  |  | *Case-control study—*Report numbers in each exposure category, or summary measures of exposure |
|  |  | 🗹 *Cross-sectional study—*Report numbers of outcome events or summary measures |
| Main results | 16 | 🗹 (*a*) Give unadjusted estimates and, if applicable, confounder-adjusted estimates and their precision (eg, 95% confidence interval). Make clear which confounders were adjusted for and why they were included |
|  |  | 🗹 (*b*) Report category boundaries when continuous variables were categorized |
|  |  | (*c*) If relevant, consider translating estimates of relative risk into absolute risk for a meaningful time period |
| Other analyses | 17 | 🗹 Report other analyses done—eg analyses of subgroups and interactions, and sensitivity analyses |
| Discussion | | |
| Key results | 18 | 🗹 Summarise key results with reference to study objectives |
| Limitations | 19 | 🗹 Discuss limitations of the study, taking into account sources of potential bias or imprecision.  🗹 Discuss both direction and magnitude of any potential bias |
| Interpretation | 20 | 🗹 Give a cautious overall interpretation of results considering objectives, limitations, multiplicity of analyses, results from similar studies, and other relevant evidence |
| Generalisability | 21 | 🗹 Discuss the generalisability (external validity) of the study results |
| Other information | | |
| Funding | 22 | 🗹 Give the source of funding and the role of the funders for the present study and, if applicable, for the original study on which the present article is based |

🗹 The sections were applicable to this study

*Give information separately for cases and controls in case-control studies and, if applicable, for exposed and unexposed groups in cohort and cross-sectional studies.
